# Validation of the German version of the late adolescence and young adulthood survivorship-related quality of life measure (LAYA-SRQL)

**DOI:** 10.1186/s12955-017-0827-1

**Published:** 2018-01-04

**Authors:** Diana Richter, Anja Mehnert, Florian Schepper, Katja Leuteritz, Crystal Park, Jochen Ernst

**Affiliations:** 1 Department of Medical Psychology and Medical Sociology, University Medical Center Leipzig, Philipp-Rosenthal-Str. 55, 04103 Leipzig, Germany; 2 Department of Pediatric Oncology, Hematology and Hemostaseology, University Medical Center Leipzig, Liebigstr. 20a, 04103 Leipzig, Germany; 3 Department of Psychology, University of Connecticut, Psychological Sciences Department, Bousfield Psychology Building, 406 Babbidge Road, Unit 1020, Storrs, CT 06269 USA

**Keywords:** Cancer, Assessment, Quality of life, Adolescents and young adults, Validation

## Abstract

**Background:**

Cancer has adverse effects on patient’s quality of life. As such, measuring quality of life (QoL) has become an integral part of psycho-oncological health care. Because adolescent and young adult patients have different needs in contrast to children and older cancer patients, instruments for adequately measuring QoL of cancer survivors in this age range are essential. As there is not a corresponding instrument in Germany, we aimed to validate the German version of the Late Adolescence and Young Adulthood Survivorship-Related Quality of Life Measure (LAYA-SRQL), a 30-item questionnaire covering 10 dimensions related to QoL.

**Methods:**

The LAYA-SRQL was translated into German following state-of-the-art criteria. We enrolled 234 adolescent and young adult (AYA) cancer patients with different tumour entities aged between 16 and 39 years old. Factorial structure was tested using confirmatory factor analysis. Internal consistency was determined by Cronbach’s α. The Short Form Survey quality of life questionnaire (SF-12v2) was used to examine convergent validity.

**Results:**

The 10-factor structure of the LAYA-SRQL was confirmed in the German sample, and the model shows high values of fit indicators: χ^2^ = 723.32 (df = 360, *p* < 0.001), CFI = 0.92, TLI = 0.90, SRMR = 0.074, RMSEA = 0.066). Subscales showed acceptable to excellent internal consistencies with Cronbach’s α > 0.70 and total Cronbach’s α of 0.93. Convergent validity was demonstrated by high positive correlations between the LAYA-SRQL and the physical (*r* = 0.45) and mental component (*r* = 0.65) of the SF-12v2.

**Conclusions:**

The German version of the LAYA-SRQL showed good psychometric properties. The instrument proved to be a highly reliable and valid instrument that can be recommended for use in the follow-up care of AYAs and for clinical research.

## Background

Every year in Germany about 15,000 people between the ages of 15 to 39 are diagnosed with cancer, compromising about 3% of all new cases. The 5-year survival rate in this group is presently approximately 80% [1]. According to the National Comprehensive Cancer Network definition (NCCN) [2] this age group is referred to as “adolescent and young adults” (AYA), although the exact ages represented in this field of research vary greatly.

AYAs represent a specific age group, which, in medical and psycho-social regards, is distinct from children and middle-aged as well as older adult (40+) cancer patients. They are at a time in life characterised by unique and complex physical, cognitive, and psycho-social developmental tasks [3]. Having cancer presents the affected person with many changes and challenges that collide with their current developmental tasks. The disease can decelerate their development and result in their original goals having to be discarded in the short term or long-term. In a recent systematic review by Quinn et al. [4] about QoL in AYA cancer patients, nearly 35 studies concluded, that AYA cancer survivors are more likely to have poorer QoL compared with the age-matched general population and older cancer survivors.

Consequences of the disease and its treatment might lead to prolonged or new dependency on parents or a lengthy interruption of education, training, or professional life and financial problems. People in this age group are doubly burdened by the challenging but ordinary developmental tasks every young person faces and the difficulties of coping with a life-threatening illness [5]. Thus, the time after treatment is often the most difficult phase of the disease because survivors have to simultaneously pursue their personal goals while also overcoming long-term consequences of their illness such as, for example, infertility caused by chemotherapy, fears of recurrence, and so on [6–8].

In the AYA-HOPE survey [9], 35% of all patients indicated at least one unsatisfied support need (e.g. psychological counselling or pain management) and more than 50% of the 524 AYAs surveyed had information needs concerning the long-term effects of the disease which were not addressed during the course of their treatment [10]. Galan and colleagues [11] identified some areas of need shared by both younger and older cancer patients; however they also found others that were specific to AYA cancer patients, such as fertility, sexual health, health behaviour, social support, and relationships with their peers. The lack of information about psychological support services was found to be associated with lower quality of life [12, 13]. This lack of information can significantly affect patients’ quality of life even several years after they have finished treatment [12, 14, 15]. Little data exists on AYA cancer survivors’ quality of life. This is due to the fact that the focus of prior studies has been on quality of life issues affecting the older cancer patient, and to the fact that adequate assessment instruments had not been developed to replicate quality of life in the context of a cancer disease specifically for this age group [4, 13]. Quality of life is, however, of central importance to the development of targeted group-specific support services and the assessment of their effectiveness. Such services could address issues like vocational rehabilitation or dealing with limited physical (e.g. fertility, sexuality) or cognitive functions [4, 8, 10].

Numerous instruments have been developed to measure health-related quality of life in cancer patients; however, we agree with other colleagues that they are often not suitable for the assessment of age-specific issues in young adult cancer patients [16–18]. Studies on quality of life in AYAs previously used non-specific assessment tools, such as the SF-36 or the EORTC QLQ-C30, which are limited to general dimensions of quality of life and do not account for age-specific issues such as fertility, body-image, or employment challenges [19–21]. Crystal Park and colleagues have therefore developed the Late Adolescence and Young Adulthood Survivorship-related Quality of Life measure (LAYA-SRQL) with a view to understanding the needs of AYAs vis-à-a their unique place in the life course and improving both medical and psychosocial care in a targeted manner [22]. Clearly defined treatment consequences and efficient screening would help to facilitate the provision of interdisciplinary care. To the best of our knowledge, there are currently no validated standard instruments in Germany for evaluating health-related quality of life in young adult cancer patients and no other quality of life measurements for young cancer patients has been developed in Germany. Considering that German is the most spoken native language in the European Union [23], there is a need for a validated tool measuring quality of life German-speaking patients aged 15 and 39 years.

The aim of the present study was to formally translate and cross-culturally adapt the German version of the LAYA-SRQL on a sample of outpatient AYA cancer patients and to test the German version for psychometric properties in terms of its reliability and validity.

## Methods

### Patients

The sample was recruited at the oncological and paediatric oncological wards of the Leipzig University Hospital from May to December 2016. Two other hospitals supported the study by delivering the questionnaire or a flyer to eligible patients. We contacted former patients by post or e-mail with a letter including written information about the study. Patients had the option of completing the survey either online or with pencil and paper. A homepage was created for the study and advertised in local newspapers, social media, and the newsletters of cancer-specific organizations. Inclusion criteria were: 1) being between 15 and 39 years old, 2) after acute treatment, and 3) having been diagnosed with cancer in the past ten years. All of the participants signed written informed consent forms or agreed to participate by ticking the box after reading the study information and the informed consent form. Minors require the signature of a parent in the paper-pencil questionnaire. We obtained approval (number 082–16-14,032,016) for the study from the local ethics committee at the University of Leipzig’s Medical Faculty.

### Measures

#### Laya-Srql

The LAYA-SRQL is a 30-item instrument for assessing health-related quality of life in the AYA cancer patients/survivors age group. Originally, the questionnaire covers two domains (satisfaction and impact) each consisting of ten dimensions with three items: 1) Existential/spirituality; 2) Coping; 3) Dependence; 4) Vitality; 5) Health Care; 6) Intimacy/sexuality; 7) Cognition/memory; 8) Relationship; 9) Education/career; and 10) Fertility. Participants indicate their level of satisfaction using a 7-point Likert scale (1 = completely unsatisfied; 2 = unsatisfied; 3 = slightly unsatisfied; 4 = neither satisfied/nor unsatisfied; 5 = slightly satisfied; 6 = satisfied; 7 = completely satisfied) and also the impact of the cancer experience (1 = very negative impact; 2 = negative impact; 3 = slightly negative impact; 4 = no impact; 5 = slightly positive impact; 6 = positive impact; 7 = very positive impact). Patients could also select “not applicable” for any item. After generating the items, the English original questionnaire was tested on a sample of *n* = 292 AYA patients between 15 and 39 years in Connecticut, USA. Internal consistencies ranged from α = 0.62 to α = 0.93. Park et al. reported good convergent validity. The 30-item model of the satisfaction-scale reached the required fit. But the model of the impact scale did not fit the data well. Both scales can be used independently in terms of their content, so we decided to exclude the impact scale from validation.

#### SF-12v2

The German Socio-Economic Panel Study (SOEP) is a representative longitudinal study of private households in Germany which surveyed annually the same private households since 1984. This questionnaire is a modified version of the SF-12 and contains 12 Items for assessing health-related quality of life with good psychometric properties [24]. In contrast to the original SF-12, the SF12v2 we used has another order. Additionally, the formulation of the items was changed and the item “During the past 4 weeks, how much did pain interfere with your normal work?” was replaced with the item “How much bodily pain have you had during the past 4 weeks?” The Socio-Economic Panel (SOEP)-Version of the Short Form Health Survey SF-12 (SF12v2) was used to assess convergent validity. The authors of the original questionnaire have also assessed convergent validity using the SF-36, so we decided to use this questionnaire correspondingly to enhance comparability. Sociodemographic data and information about cancer diagnosis and oncological treatment were obtained via self-report.

### Translation process

The LAYA-SRQL was translated into German following state-of-the-art criteria using a forward-backward-translation method. The translation from the original questionnaire into German was performed by two independent translators. These two translations were synthesised to a single German version after discussion. A back-translation of the synthesised version was performed by two independent native speakers who had never seen the original questionnaire. The backward-translation was compared with the original questionnaire, and after discussing discrepancies, a final consensus version of the questionnaire was created. We pre-tested the questionnaire with the cognitive interviewing method to identify problematic questions. For this step, five haematological patients completed the questionnaire and were interviewed afterward according to the 4-factor model of Tourangeau, the four factors being comprehension of a question, retrieval of information from memory, the judgment or estimation process, and the final selection of a response to the question [25, 26].

### Statistical analyses

Item analysis comprised means, standard deviations, discriminatory power, skewness and kurtosis as well as factor loadings. A confirmatory factor analysis (CFA) was conducted to test the model. The model fit provided the Comparative Fit Index (CFI), the Tucker–Lewis Index (TLI), Root-Mean-Square Error of Approximation (RMSEA), χ2 over degrees of freedom and Standardized Root Mean Residual (SRMR) which is suggested by Hu & Bentler [27]. We analysed “not applicable” (N/A) responses for each item. N/A responses were treated as missing. Missing data were entered using stochastic regression according to the original questionnaire. Means, standard deviation and ceiling and floor effects were calculated for each dimension. According to McHorney et al., ceiling and floor effects occur when >20% of the participants indicate highest or lowest scores [28]. Reliability analyses comprised the average variance extracted (AVE), the standard error of measurement (SEM) and the composite reliability (CR). The internal consistency of the subscales was also computed using Cronbach’s alpha coefficient. Pearson correlations between the LAYA-SQRL and the SF-12v2 were used to assess convergent validity. Hierarchical regression analysis was used to control for age and gender with the dimensions of the LAYA-SRQL as dependent variable and the physical component summary score (PCS) and mental component summary score (MCS) as independent variables. Data analyses were conducted using SPSS 24.0 and AMOS 23.0 software.

## Results

### Sample

A total of *n* = 234 young cancer patients between 16 and 39 years old with a median of 30.0 years at the time of the survey were enrolled. Participants were mostly single and childless. The majority had a haematological cancer diagnosis. Time since diagnosis had a median of 34.0 (IQR = 42.5) months. Detailed patient characteristics are shown in Table 1.

**Table 1:** Sample demographic and medical characteristics (*n* = 234)

		n	%
Age in years (median [interquartile range])		30.0 (9)	
Gender	Female	186	79.5
	Male	48	20.5
Marital status	Married	61	26.2
	Single	165	70.5
	Divorced	8	3.4
Partnership/cohabiting (yes)		150	64.1
Children (yes)		48	20.5
Educational level	High school degree	173	73.9
	Secondary school degree	51	21.8
	Junior high school degree	5	2.2
	No educational degree	5	2.1
Employment status^1^	Employed, fulltime	97	42.7
	Employed, part-time	55	24.2
	Unemployed/Students	75	33.1
Off treatment (yes)		187	79.9
Sick leave (yes)		42	17.9
Tumor site	Hematological malignancies	125	53.4
	Breast	57	24.4
	Sarcoma	13	5.6
	other solid tumors	39	16.6
Time since diagnosis in months (median [interquartile range])		34.0 (42.5)	
Cancer treatments (multiple responses possible)	Surgery	128	54.7
	Radiation therapy	137	58.5
	Chemotherapy	212	90.6
	Bone marrow/stem cell transplantation	39	16.7
	Hormonal therapy	42	17.9

^1^missing value: *n* = 7 (valid percentages shown)

### Item analysis

Factor loadings, means, SD, discriminatory power, skewness and kurtosis are displayed in Table 2. Discriminatory power ranged from 0.39 to 0.85. The item “My ability to concentrate” showed the highest coefficients and the item “My health coverage” the lowest. Mean scores of the items ranged from 2.61 to 5.36 with the midpoint 4, and mean scores of the subscales ranged from 3.03 (“Fertility”) to 5.25 (“Relationship”). All factor loadings were >0.50 (range 0.51–0.98). Each subscale correlated positively with the other subscales (Table 3). The significant intercorrelations of the subscales revealed strong construct validity (Table 4).

**Table 2:** Item and scale characteristics

		Item characteristics						Scale characteristics					
No.	Scales and items	M	SD	r i(t-i)	Skewness	Kurtosis	Factor loading	Cronbach’s α	Floor effect (%)	Ceiling effect (%)	CR	SEM	AVE
	Intimacy/Sexuality							0.91	9.83	8.12	0.91	0.09	0.76
15	My desire for physical intimacy	3.89	1.86	0.82	0.11	−1.08	0.88						
5	My comfort with physical intimacy.	3.86	1.92	0.81	0.19	−1.19	0.87						
16	My sexual functioning.	3.97	1.87	0.81	0.14	−1.16	0.87						
	Cognition/Memory							0.90	8.55	3.84	0.90	0.10	0.75
21	My ability to concentrate.	3.40	1.80	0.85	0.45	−1.02	0.91						
14	My short-term memory.	3.44	1.81	0.81	0.43	−0.88	0.88						
27	My ability to think clearly.	4.10	1.86	0.73	−0.00	−1.27	0.81						
	Fertility							0.84	17.95	2.99	0.86	0.14	0.68
4	My ability to have children.	2.95	1.74	0.83	0.77	0.12	0.98						
6	My concerns about fertility.	2.61	1.53	0.68	0.86	0.46	0.78						
19	My desire to have children.	3.53	1.85	0.63	0.29	−0.86	0.68						
	Relationship							0.78	0	13.25	0.82	0.16	0.62
13	My compassion for others.	5.27	1.45	0.70	−0.88	0.13	0.85						
30	My ability to empathize with others.	5.36	1.34	0.74	−0.99	0.49	0.94						
17	My ability to share my experiences with others	5.13	1.56	0.46	−0.61	−0.62	0.51						
	Education/Career							0.82	1.28	10.26	0.85	0.15	0.65
18	My desire to educate myself.	4.84	1.47	0.78	−0.22	−0.74	0.89						
3	My interest in learning new things.	4.74	1.62	0.71	−0.33	−0.99	0.88						
24	My educational and career goals.	4.32	1.80	0.56	−0.26	−1.02	0.63						
	Vitality							0.84	8.12	4.27	0.86	0.14	0.67
26	My ability to participate in my favourite sports and hobbies.	4.29	1.93	0.73	−0.23	−1.32	0.83						
23	My ability to exercise.	4.07	1.96	0.79	−0.03	−1.38	0.90						
2	My energy level.	3.29	1.75	0.61	0.43	−1.02	0.71						
	Health care							0.72	1.28	5.14	0.74	0.26	0.51
20	My health coverage.	4.66	1.66	0.39	−0.34	−0.77	0.40						
10	My ability to obtain health care.	4.81	1.55	0.66	−0.46	−0.84	0.76						
9	My sense of security that my health care needs will be met.	4.29	1.62	0.58	−0.12	−1.16	0.89						
	Dependence							0.76	1.28	2.99	0.78	0.22	0.54
1	My dependence on others.	4.10	1.66	0.62	0.18	−0.76	0.78						
29	My dependence on family.	4.26	1.59	0.67	−0.01	−0.75	0.81						
11	My reliance on others.	4.65	1.68	0.48	−0.41	−0.95	0.60						
	Spirituality							0.85	1.28	4.28	0.85	0.15	0.66
7	My engagement in religious and/or spiritual practices.	4.42	1.38	0.72	−0.09	0.28	0.82						
8	My spiritual life.	4.61	1.33	0.76	−0.21	−0.36	0.87						
28	My sense of closeness to God or a higher power.	4.28	1.43	0.67	−0.34	−0.05	0.74						
	Coping							0.83	7.26	2.99	0.83	0.17	0.62
12	My ability to manage stress.	4.02	1.88	0.69	−0.10	−1.31	0.78						
22	My sense of control over my life.	3.45	1.77	0.65	0.33	−1.10	0.78						
25	My ability to relax.	4.05	1.83	0.73	0.00	−1.20	0.81

**Table 3:** Subscale intercorrelations LAYA-SRQL (*n* = 234)

Subscale	Intimacy	Cognition	Fertility	Education	Vitality	Health Care	Relationship	Dependence	Spirituality	Coping
Intimacy	1									
Cognition	0.47***	1								
Fertility	0.37***	0.33***	1							
Education	0.43***	0.56***	0.24***	1						
Vitality	0.42***	0.58***	0.23***	0.55***	1					
Health Care	0.40***	0.36***	0.23***	0.32***	0.31***	1				
Relationship	0.36***	0.35***	0.15*	0.44***	0.30***	0.32***	1			
Dependence	0.41***	0.50***	0.23***	0.50***	0.48***	0.45***	0.42***	1		
Spirituality	0.32***	0.19**	0.25***	0.35***	0.30***	0.32***	0.47***	0.42***	1	
Coping	0.46***	0.67***	0.31***	0.63***	0.60***	0.51***	0.44***	0.64***	0.37***	1
Total satisfaction scale	0.70***	0.76***	0.50***	0.74***	0.72***	0.61***	0.60***	0.73***	0.56***	0.84***

***p < 0.001; **p < 0.01; *p < 0.05

**Table 4:** Scale Intercorrelations with SF12v2 (*n* = 226)

	Intimacy	Cognition	Fertility	Education	Vitality	Health Care	Relationship	Dependence	Spirituality	Coping	Total satisfaction scale
SF-12v2											
Physical component	0.27***	0.37***	0.26***	0.28***	0.57***	0.33***	0.06	0.26***	0.06	0.32***	0.45***
Physical functioning	0.37***	0.44***	0.25***	0.34***	0.58***	0.38***	0.13	0.37***	0.08	0.37***	0.53***
Role physical	0.34***	0.50***	0.28***	0.40***	0.61***	0.34***	0.19*	0.33***	0.16*	0.46***	0.55***
Bodily Pain	0.24***	0.36***	0.19**	0.29***	0.46***	0.36***	0.11	0.30***	0.06	0.41***	0.42***
General Health	0.37***	0.43***	0.32***	0.41***	0.57***	0.41***	0.22**	0.33***	0.21**	0.49***	0.56***
Mental component	0.45***	0.56***	0.23***	0.54***	0.50***	0.44***	0.41***	0.50***	0.28***	0.66***	0.65***
Vitality	0.42***	0.56***	0.23**	0.47***	0.64***	0.36***	0.28***	0.42***	0.20**	0.54***	0.61***
Social functioning	0.41***	0.46***	0.29***	0.50***	0.58***	0.40***	0.29***	0.44***	0.25***	0.56***	0.61***
Role emotional	0.43***	0.54***	0.21**	0.47***	0.47***	0.46***	0.36***	0.48***	0.21**	0.60***	0.61***
Mental health	0.42***	0.52***	0.25***	0.50***	0.45***	0.46***	0.38***	0.47***	0.25***	0.63***	0.62***

*p < 0.05; **p < 0.01; ***p < 0.001

### Factorial structure and scale characteristics

A confirmatory factor analysis was performed to test whether the ten domains of the original questionnaire can describe the data of the German version sufficiently. The model reached an acceptable model (χ^2^ = 723.32 (360), *p* < 0.001; χ^2^/*df* = 2.01; CFI = 0.92, TLI = 0.90, SRMR = 0.074, RMSEA = 0.066).

A high reliability of the whole questionnaire (α = 0.93) was found using Cronbach’s Alpha coefficient. The subscales showed good internal consistency, between α = 0.72 and α = 0.91 (Table 2). Also, Composite reliability and average variance extracted of the domains ranged from 0.74 to 0.91 and from 0.51 to 0.76, respectively and showed good reliability with low SEM (range 0.09–0.26; Table 2). The scales showed no significant floor or ceiling effects >20%.

### Convergent validity

Convergent validity testing revealed a significantly positive relationship with the SF-12v2 quality of life measurement tool. Table 5 contains the correlations between the LAYA-SRQL satisfaction scale and the SF-12v2 subscales and the sum scores of PCS and MCS of the SF-12v2. Regression analyses revealed also a strong impact of the dimensions of the LAYA-SRQL on the PCS and MCS. No effects of gender and age could be found.

### Missing values

The items of the dimension “Spirituality” had the most “not applicable” responses. Nearly half of the participants (47.9%) rated the item “My engagement in religious and/or spiritual practices” as “not applicable”, 42.7% the item “My sense of closeness to God or a higher power”, and 41% the item “My spiritual life”. After the dimension “Spirituality”, all items of the dimension “Fertility” had the most “not applicable” responses.

## Discussion

In order to ascertain the support needs of the AYA-group and to reduce the long-term impact of cancer on this population, there is an urgent need for a target group-specific, valid, and practical assessment tool that can be implemented within the context of follow-up care. The LAYA-SRQL is a valid and reliable questionnaire for assessing relevant quality of life domains among adolescent and young adult cancer survivors. So far, there has not been an equivalent instrument within the German-speaking countries. Therefore, the presented study aimed to describe the translation and validation of the age-specific LAYA-SRQL.

The data supported the reliability and validity of the German version as well as the replication of the 10-factorial structure of the original questionnaire in our sample.

Internal consistencies of all eight subscales demonstrated good reliability with Cronbach’s alpha >0.7 and also CR > 0.7. In contrast to the original scales, internal consistencies in the German version were lower except for the “Education” and “Health Care” dimensions. As already described in the original LAYA-SRQL, the German sample reported being most satisfied with “Relationship” and “Education”. The dimension of “Health Care” showed better means in the German sample. This may be due to the differences between the German and the American health care system. Participants in both samples were most dissatisfied with issues encompassed by the “Fertility”, “Intimacy”, and “Cognition” dimensions, a finding that has emerged in previous studies as well [14, 29]. Our results show that the German LAYA-SRQL strongly correlated with the SF-12v2 tool for measuring health-related quality of life, which confirmed the convergent validity of the scale.

Along with the value of these results, this study does have some limitations. Due to the cross-sectional study design we were not able to measure test-retest reliability. Further studies should examine this issue.

Furthermore, we could not conduct non-responder analyses because of the different ways participants could access the study and the fact that the cooperating hospitals did not document their recruitment efforts in detail. Strengths of the study include its sample size since the AYAs were difficult to recruit and the low rate of missing values for conducting confirmatory factor analysis.

Additionally, the high number of “not applicable” answers of the dimension “Spirituality” has to be considered in future research. This fact might have its roots in a cultural pattern; thus, the cultural environment must be considered when measuring spirituality [30]. Spirituality (according to a broader concept of religion) has a higher level of importance in the USA than in Germany [31]. Cross-cultural research between the USA and Germany revealed that self-identifying as being “spiritual” or “religious” is much more common in the USA [32, 33]. There are many more widely recognized spiritual practices, religions, and denominations of Christian churches in the USA than in Germany allowing for more individualized selections of a religious community. The USA is an example of the religious market model in terms of the theory of pluralization in contrast to the theory of secularization, which is predominant in Germany [34]. The Protestant community and the Catholic community are the two major churches in Germany. Most German members of a religious community were raised in religious homes and they rarely change their religion. The German churches are more secularized (e.g. with the implementation of church tax) and therefore the construct cannot be transferred to the German context [33, 35]. Also, according to surveys, even 27 years after German reunification half of the people in former East Germany describe themselves as not being religious or spiritual in contrast to people from former West Germany [36, 37]. But we cannot make any statement according to the socialization in our sample, because we have only asked where the participants live at the time of the survey and not where they grew up or were socialized.

There have been various studies from Anglo-American regions that have measured spirituality within the context of health-related quality of life [38–40]. And some measurements have included spirituality as a psychological or social domain such as the MHIQ or the WHQOL-100 [41, 42].

Although a link has been found between quality of life and spirituality, little is known about the causal direction of this association. Previous research indicates positive effects of spirituality on quality of life especially for older cancer patients and patients in palliative treatment [43–46]. So far though, there has been a paucity of research investigating the impact of spirituality on quality of life in adolescent and young adult cancer patients. We therefore recommend assessing the “spiritual quality of life” based on additional modules of quality of life measures (e.g. FACIT-Sp [47] or WHOQOL-SRPB [48]). But there is ongoing controversy in research whether to integrate or exclude spirituality and religion in the concept of health-related quality of life [49].

Additionally, further research should investigate the application of the German scale in clinical practice with a larger sample to enable comparisons between several tumour entities. Because, as already mentioned by Park et al. [22], some issues like fertility may not be relevant to all AYAs (e.g. the adolescents and younger adults) longitudinal studies with a broader age range should be conducted to compare quality of life issues between age subgroups within the AYA population and to observe developments according to treatment related sequelae.

## Conclusions

In summary, the presented data support a valid and reliable instrument for measuring health-related quality of life that provides valuable information for physicians in the management with young cancer patients.

Good psychometric properties confirmed the use of the German satisfaction scale of the LAYA-SRQL in psychooncological research of the AYA population. Future studies should consider reducing the scale of this assessment tool for more economical use in clinical follow-up care.

## Abbreviations

AVE: Average variance extracted
AYA: Adolescent and young adults
CFA: Confirmatory factor analysis
CFI: Comparative Fit Index
CR: Composite reliability
EORTC QLQ-C30: The European Organization for Research and Treatment of Cancer Quality of Life core questionnaire C30
FACIT-Sp: Functional Assessment of Chronic Illness Therapy - Spiritual Well-Being
IQR: Interquartile range
LAYA-SRQL: Late Adolescence and Young Adulthood Survivorship-related Quality of Life measure
MCS: Mental component summary score
MHIQ: McMaster Health Index Questionnaire
NCCN: National Comprehensive Cancer Network definition
PCS: Physical component summary score
QoL: Quality of Life
RMSEA: Root-Mean-Square Error of Approximation
SEM: Standard error of measurement
SF12v2: Socio-Economic Panel (SOEP)-Version of the SF-12
SF-36: Short Form Health Survey-36
SOEP: German Socio-Economic Panel Study
SRMR: Standardized Root Mean Residual
TLI: Tucker–Lewis Index
WHOQOL-SRPB: World Health Organization Quality of Life Group-Spirituality, Religion and Personal Beliefs questionnaire
WHQOL-100: World Health Organization Quality of Life Group-100
