# Correction to: Validation of the German version of the late adolescence and young adulthood survivorship-related quality of life measure (LAYA-SRQL)

**DOI:** 10.1186/s12955-018-0852-8

**Published:** 2018-01-30

**Authors:** D. Richter, A. Mehnert, F. Schepper, K. Leuteritz, C. Park, J. Ernst

**Affiliations:** 1 Department of Medical Psychology and Medical Sociology, University Medical Center Leipzig, Philipp-Rosenthal-Str. 55, 04103 Leipzig, Germany; 2 Department of Medical Psychology and Medical Sociology, University Medical Center Leipzig, Philipp-Rosenthal-Str. 55, 04103 Leipzig, Germany; 3 Department of Pediatric Oncology, Hematology and Hemostaseology, University Medical Center Leipzig, Liebigstr. 20a, 04103 Leipzig, Germany; 4 Department of Psychology, University of Connecticut, Psychological Sciences Department, Bousfield Psychology Building, 406 Babbidge Road, Unit 1020, Storrs, CT 06269 USA

## Erratum

The original article contained the following errors:

1. On Page 2, the sub-heading ‘LAYA-SRQL’ was un-capitalised.
2. On Page 4, the sentence, “The significant intercorrelations of the subscales revealed strong construct validity (Table 3).” incorrectly made reference to Table 4.
3. On Page 4, the sentence, “Table 4 contains the correlations between the LAYA-SRQL satisfaction scale and the SF-12v2 subscales and the sum scores of PCS and MCS of the SF-12v2.” incorrectly made reference to Table 5.

The original article [1] has now been updated to correct these errors; furthermore, these errors were the fault of the Production team carrying this article forward, and as such, these errors were not the fault of the author.
